# Correction: Derivation and validation of a clinical prediction model for risk-stratification of children hospitalized with severe pneumonia in Bangladesh

**DOI:** 10.1371/journal.pgph.0002412

**Published:** 2023-09-14

**Authors:** The PLOS Global Public Health Staff

## Notice of republication

This article was republished on August 30, 2023, to correct the affiliation of the editor, which incorrectly was typeset as the authors’. The publisher apologizes for the errors. Please download this article again to view the correct version. The originally published, uncorrected article and the republished, corrected articles are provided here for reference.

## Supporting information

S1 File Originally published, uncorrected article. (PDF)

S2 File Republished, corrected article. (PDF)
