# Supplementary material for: Correction: Derivation and validation of a clinical prediction model for risk-stratification of children hospitalized with severe pneumonia in Bangladesh
Source: PLOS Glob Public Health. 2023 Sep 14;3(9):e0002412. doi: 10.1371/journal.pgph.0002412 (PMC10501663; doi:10.1371/journal.pgph.0002412)
Supplement: S2 File — (PDF) [file pgph.0002412.s002.pdf]

PLOS GLOBAL PUBLIC HEALTH

RESEARCH ARTICLE

Derivation and validation of a clinical prediction model for risk-stratification of children hospitalized with severe pneumonia in Bangladesh

Gazi Md. Salahuddin Mamun[1‡], Michael Zou[2‡], Monira Sarmin[3], Ben J. Brintz[4], Abu Sayem Mirza Md. Hasibur Rahman[3], Irin Parvin[3], Mst Mahmuda Ackhter[3], Mohammod Jobayer Chisti[3], Daniel T. Leung[2,5‡], Lubaba Shahrin[3‡*]

1 Infectious Diseases Division, International Centre for Diarrhoeal Disease Research, Bangladesh (icddr,b), Dhaka, Bangladesh, **2** Division of Infectious Diseases, University of Utah School of Medicine, Salt Lake City, Utah, United States of America, **3** Nutrition and Clinical Services Division, International Centre for Diarrhoeal Disease Research, Bangladesh (icddr,b), Dhaka, Bangladesh, **4** Division of Epidemiology, University of Utah School of Medicine, Salt Lake City, Utah, United States of America, **5** Division of Microbiology & Immunology, University of Utah School of Medicine, Salt Lake City, Utah, United States of America

‡ GMSM and MZ are joint first authors on this work. DTL and LS are joint senior authors on this work.
* lubabashahrin@icddrb.org

Check for updates

OPEN ACCESS

Citation: Mamun GM.S, Zou M, Sarmin M, Brintz BJ, Rahman ASMM.H, Parvin I, et al. (2023) Derivation and validation of a clinical prediction model for risk-stratification of children hospitalized with severe pneumonia in Bangladesh. PLOS Glob Public Health 3(8): e0002216. https://doi.org/10.1371/journal.pgph.0002216

Editor: Sindhu Kulandaipalayam Natarajan, Christian Medical College Vellore, INDIA

Received: February 15, 2023

Accepted: July 6, 2023

Published: August 1, 2023

Copyright: © 2023 Mamun et al. This is an open access article distributed under the terms of the Creative Commons Attribution License, which permits unrestricted use, distribution, and reproduction in any medium, provided the original author and source are credited.

Data Availability Statement: All data can be found in the manuscript and supporting information files.

Funding: The authors received no specific funding for this work.

Competing interests: The authors have declared that no competing interests exist.

Abstract

Children with severe pneumonia in low- and middle-income countries (LMICs) suffer from high rates of treatment failure despite appropriate World Health Organization (WHO)-directed antibiotic treatment. Developing a clinical prediction rule for treatment failure may allow early identification of high-risk patients and timely intervention to decrease mortality. We used data from two separate studies conducted at the Dhaka Hospital of the International Centre for Diarrheal Disease Research, Bangladesh (icddr,b) to derive and externally validate a clinical prediction rule for treatment failure of children hospitalized with severe pneumonia. The derivation dataset was from a randomized clinical trial conducted from 2018 to 2019, studying children aged 2 to 59 months hospitalized with severe pneumonia as defined by WHO. Treatment failure was defined by the persistence of danger signs at the end of 48 hours of antibiotic treatment or the appearance of any new danger signs within 24 hours of enrollment. We built a random forest model to identify the top predictors. The top six predictors were the presence of grunting, room air saturation, temperature, the presence of lower chest wall indrawing, the presence of respiratory distress, and central cyanosis. Using these six predictors, we created a parsimonious model with a discriminatory performance of 0.691, as measured by area under the receiving operating curve (AUC). We performed external validation using a temporally distinct dataset from a cohort study of 191 similarly aged children with severe acute malnutrition and pneumonia. In external validation, discriminatory performance was maintained with an improved AUC of 0.718. In conclusion, we developed and externally validated a parsimonious six-predictor model using random forest methods to predict treatment failure in young children with severe pneumonia in Bangladesh. These findings can be used to further develop and validate parsimonious and pragmatic prognostic clinical prediction rules for pediatric pneumonia, particularly in LMICs.

Introduction

Despite advances in modern medicine, pneumonia remains among the leading causes of mortality in children under 5, with annual deaths estimated at 740,000 [1]. Pneumonia alone accounts for 15% of an estimated 5.4 million global deaths in children under 5 years of age [2]. As a result, a recent report commissioned by the WHO emphasized the importance of further research in the risk stratification of children with pneumonia [3].

The greatest burden of pneumonia is concentrated in low- and middle-income countries (LMICs), where resources for medications and hospital-based management are limited [4]. The rates of treatment failure are considerably higher in LMICs compared to high-income countries [5]. A randomized controlled trial on under-five children conducted in a tertiary-level hospital in Bangladesh found that 14% of participants experienced treatment failure, with 53% of patients requiring switching to second-line antibiotics [6]. Another cohort study from Tanzania found that the rate of first-line antibiotic treatment failure was over 50% among children with severe community-acquired pneumonia [7]. Identifying children with a high risk of treatment failure will help to triage appropriate resources for children who need closer inpatient monitoring and post-discharge follow-up.

Prior research has identified predictors associated with treatment failure. For example, Chisti *et al.* in Bangladesh found that dehydration, hypocalcemia, and bacteremia were independently associated with treatment failure [8]. Likewise, Muro *et al.* found that in their Tanzanian facility, HIV positivity, acute malnutrition, poor oxygen saturation, convulsions, central cyanosis, and abnormal chest X-rays were independently associated with treatment failure [7]. The goal of our study is to build on this body of research by using machine learning methods on datasets of children with severe pneumonia to build a pragmatic clinical prediction model to identify children at high risk of treatment failure.

Methods

Derivation dataset

The data for the derivation of the clinical prediction rule was obtained from a recently completed randomized controlled trial comparing antibiotic regimens for the treatment of severe pneumonia in 308 children aged 2–59 months in a tertiary center in Bangladesh. Full details of the study design are described elsewhere [9, 10]. Briefly, the study enrolled patients from the Dhaka Hospital of International Centre for Diarrheal Disease Research, Bangladesh (icddr,b), an urban facility that provides care for nearly 200,000 patients a year according to the icddr,b annual report, 2021. Children aged 2–59 months were recruited from January 2018 to October 2019. The median age of the children among control and intervention groups were 7.1 and 7.2 months respectively with a male predominance (60% and 65% respectively). All participants had severe pneumonia according to the revised 2014 WHO guidelines for pediatric pneumonia [11], based on cough and difficulty in breathing, plus at least two of the following "danger signs": (i) central cyanosis or oxygen saturation <90% on pulse oximetry, (ii) severe respiratory distress (e.g., grunting, very severe chest indrawing), (iii) signs of pneumonia with a general danger sign (e.g., inability to breast-fed or drink, lethargy or unconsciousness, convulsion). Exclusion criteria included: those who received antibiotics at home or another hospital for ≥48 hours prior to admission, had known congenital or chromosomal anomaly (e.g., congenital heart disease, laryngomalacia, cleft lip, cleft palate, and trisomy 21), or required cardio-pulmonary resuscitation or referral to another facility for management of

acute renal failure. Treatment failure was defined as either persistence of danger signs (central cyanosis, hypoxemia, grunting, inability to breast-fed or drink, lethargy or convulsions) at the end of 48 hours, or the appearance of new danger signs including requiring mechanical ventilation, development of severe sepsis or septic shock, death, or referral to another specialized hospital (e.g. for acute kidney injury requiring dialysis) within 24 hours of enrollment. Among the participants, 14 (4.5%) children died [9].

External validation dataset

For the external validation of our prediction model, we used data from a cohort study of 191 children aged 0–59 months with severe acute malnutrition and severe pneumonia, hospitalized between April 2015 and March 2017 in the Dhaka Hospital of icddr,b. Clinical and demographic information was collected at enrollment, and information regarding outcomes was recorded at discharge and at 30-day follow-up from admission. The median age of the enrolled children was 8.0 months with a male predominance (61%). 78% had been born preterm. A total of 14 (7.3%) children died within 30 days of hospital admission; of these, 12 (6.3%) died in-hospital and two (1.0%) died after discharge. These deaths were coded as treatment failures for the validation of our model. Details of this study can be found elsewhere [12]. For the external validation, only parameters identified in the derivation analysis and selected for inclusion in the prediction rule were used.

Derivation of a clinical prediction rule

The derivation dataset contained patient information for 185 candidate predictors that were assessed at the time of presentation. We conducted an initial variable screening in which predictors with few positives or negatives (i.e. less than 5% of patients) were excluded. For example, 99% of participants had a cough so it would be unlikely to be helpful in a prediction model. Other reasons for initial exclusion were similarity (such as heart rate and pulse) or lack of clinical relevance. The derivation dataset had also randomized patients to amoxicillin and ampicillin arms in a 1:1 fashion. In order to create a more generalizable prediction rule, the antibiotic assignment was excluded. After this initial screening, we further analyzed 131 predictors. Missing data were imputed by a single imputation using the multiple imputations by chained equations (MICE) package in R [13].

We used repeated 5-fold cross-validation using 100 iterations to estimate the generalizable performance of the random forest model and determine the optimal number of predictors. This was done with the permimp package and cforest function in R, a conditional permutation approach that is particularly robust to co-linearity when estimating variable performance [14]. Following cross-validation, the optimal model was applied to the entire dataset. We assessed discriminatory performance by calculating the AUC (area under the receiver operating characteristic curve (ROC). The ROC curve plots the true positive rate against the false positive rate, with the AUC providing the probability that our model correctly predicted the outcome (ie. treatment failure). Calibration was assessed by using calibration-in-the-large and calibration slope [15]. External validation was performed using similar methods on the validation dataset described above [12].

For the development of the prediction models and further analysis, R was used. The TRIPOD (transparent reporting of a multivariable prediction model for individual diagnosis) guidelines were followed (S1 Text).

Ethical approval statement

This is a secondary analysis from the data of two different studies among the children. Ethical approval for both of the studies had been given by the Institutional Review Board (IRB) of International Centre for Diarrhoeal Disease Research, Bangladesh (icddr,b). Informed written consent was obtained from the legal guardian of all participants before enrolling into the studies.

Results

Fig 1 and Table 1 describe the process of developing the random forest model, with a representative sample of the types of predictors that were included. There was no missing data for the outcome of treatment failure, with 62 (20%) of patients experiencing treatment failure. Treatment failure was due to persistence of danger signs in 30 patients (10%) and development of new danger signs in 32 patients (10%). Predictors that required imputation included family income, adequacy of oral rehydration, type of diarrhea, height, mid upper arm circumference (MUAC), weight for height z-score (WHZ), height for age z-score (HAZ), pulse, room air saturation, systolic and diastolic blood pressure. For these predictors, less than 5% of patients had

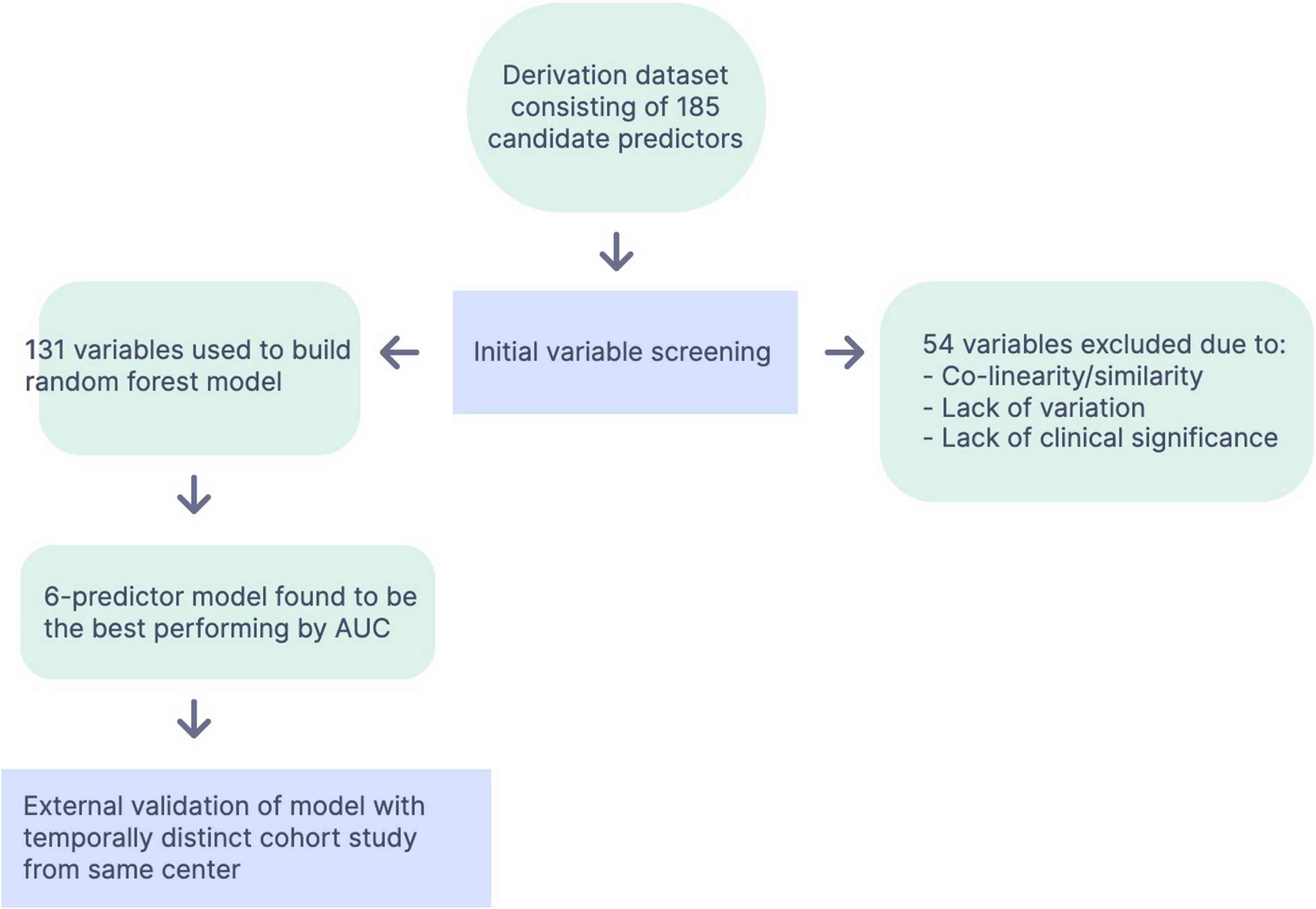

Fig 1. Process of model building.

https://doi.org/10.1371/journal.pgph.0002216.g001

Table 1. Representative sample of predictors included in prediction model.

Category of variables	Examples
Demographics and living situation	• Age and sex<br>• Family income, number of family members, education levels
Details of birth	• Birth complications such as jaundice<br>• Birth weight
Recent foods consumed	• Breast milk, cow's milk<br>• Quantity and type of oral rehydration solution consumed
Vaccination status	• Doses received of BCG, PCV, MMR
Symptoms at the time of enrollment	• Irritability<br>• Poor urine output<br>• Dyspnea
Signs at the time of enrollment	• Vital signs<br>• Height and weight adjusted for age<br>• Grunting, lethargy, nasal flaring

https://doi.org/10.1371/journal.pgph.0002216.t001

a missing value. Further description of baseline patient characteristics was published in the original study [9].

The top 10 predictors for the random forest model are provided in Table 2, with the six most important predictors being the presence of grunting (yes or no), room air saturation, temperature, presence of lower chest wall indrawing (yes or no), presence of respiratory distress (yes or no), and presence of central cyanosis (yes or no). The order of importance for these predictors did not change with the adjustment of various tuning parameters of our model, such as the number of trees included in the model. The most important predictors could be assessed during the initial physical exam. Indeed, two of the top three predictors are vital signs. Duration of poor feeding was the only predictor among the top 10 that would need to be assessed by history taking.

Partial dependency plots of the top six predictors are shown in Fig 2. These help to demonstrate the magnitude and effect direction of our top predictors on treatment failure. For example, the top predictor, the presence of grunting, was associated with a roughly 78% chance of treatment failure if it was present. Conversely, treatment failure was seen in roughly 14% of patients who did not have grunting on examination. The discriminatory power of predictors like the presence of lower chest wall indrawing was less pronounced, with 24% of those with this finding experiencing treatment failure compared to 14% of those without. The continuous

Table 2. Top 10 predictors for treatment failure. Importance is defined as the amount that the AUC would decrease if the predictor were to be excluded. For categorical variables, the number of patients positive for a given predictor are provided. For continuous variables, the median value is presented.

Variable	Importance	Number of Patients with Predictor (Percentage)
Presence of grunting	0.0246	48 (15.6)
Room air saturation	0.0081	97 percent
Temperature	0.0041	37.4 degrees Celsius
Presence of lower chest wall indrawing	0.0036	264 (85.6)
Presence of severe respiratory distress	0.0032	92 (29.8)
Presence of central cyanosis	0.0026	49 (15.9)
Weight for height z-score (WHZ)	0.0013	-1.45 standard deviations
Weight for age z-score (WAZ)	0.0012	-2.09 standard deviations
Duration of Poor Feeding	0.0012	1 day
Mid Upper Arm Circumference (MUAC)	0.0011	12.5 cm

https://doi.org/10.1371/journal.pgph.0002216.t002

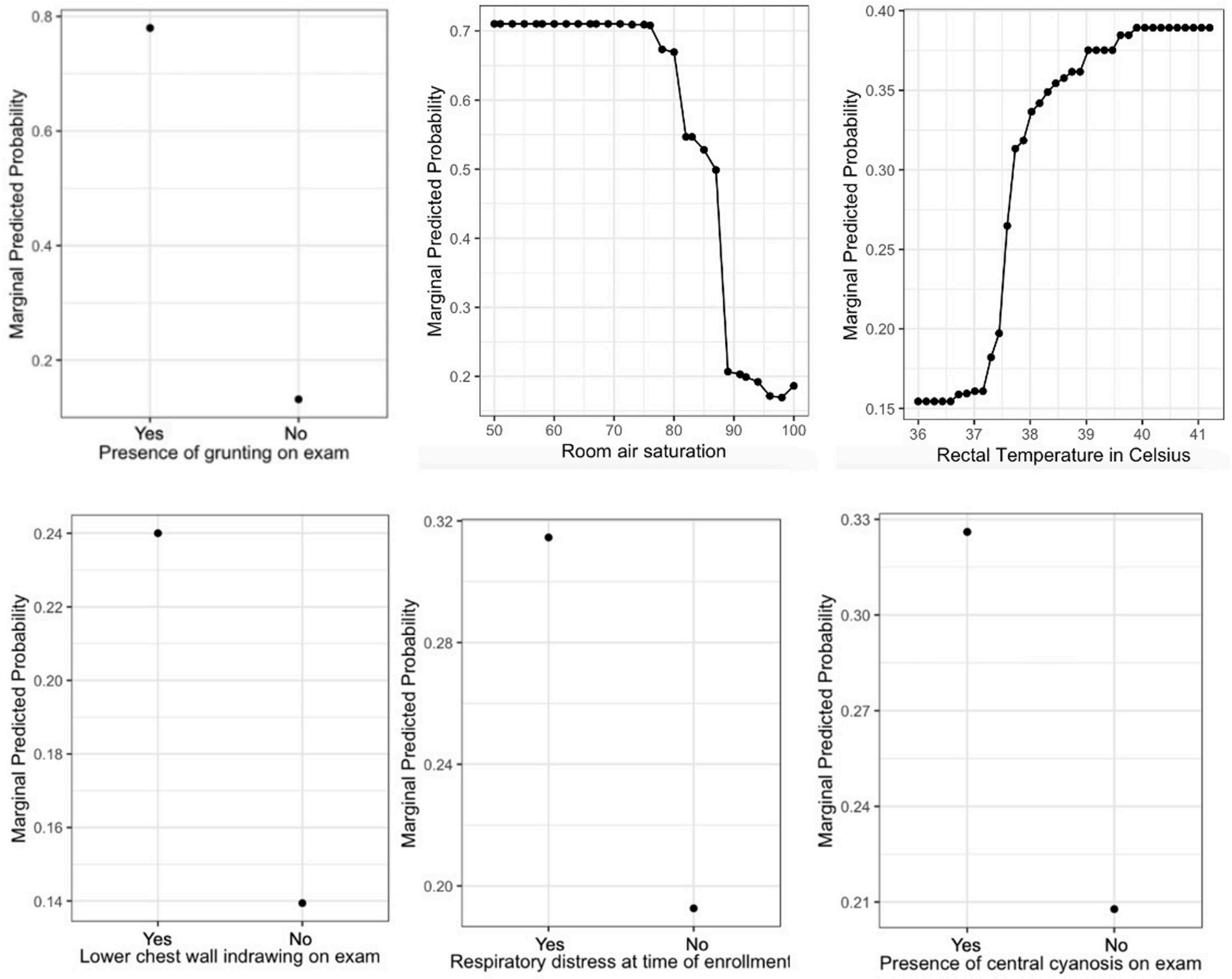

Fig 2. Partial dependency plots of the top 6 predictors.

https://doi.org/10.1371/journal.pgph.0002216.g002

variables of room air saturation and temperature demonstrated some dichotomous characteristics with sharp changes in treatment failure probability at certain values. For example, treatment failure significantly decreased in patients with room air saturations greater than 89 percent and temperatures less than 37.5 degree Celsius.

We performed repeated five-fold cross-validation with our random forest models across 100 iterations, achieving an average AUC that ranged from 0.641 (2 variables) to 0.696 (7 variables), with standard deviations ranging from 0.0686 to 0.0936 for other models using between one and ten predictors. Interestingly, increasing the number of variables beyond 7 decreased the discriminatory performance of our model. Likewise, the improvement in AUC from 1 variable to 7 variables, or certainly 3 variables (AUC of 0.663) to 7 variables was modest (Fig 3). We ultimately used a model composed of 6 predictors, as the improvement in discriminatory performance from 6 to 7 predictors (0.691 to 0.696) was too modest to justify adding an additional predictor in a pragmatic clinical prediction tool.

The 6-variable model was externally validated by applying it to a cohort study of 191 children with severe pneumonia from Dhaka Hospital between 2015 and 2017, which examined

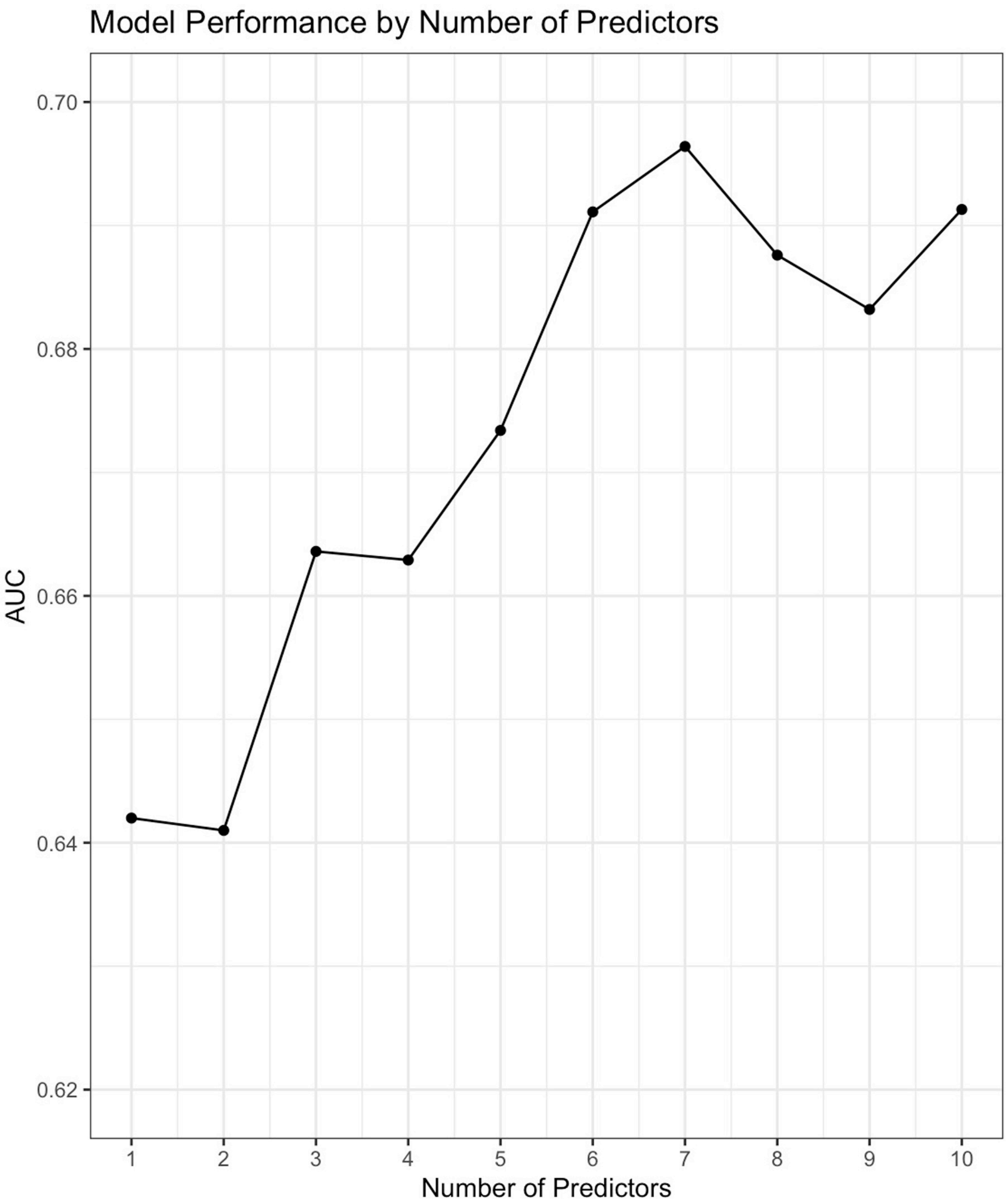

Fig 3. Model performance according to number of variables by 5-fold cross-validation.

https://doi.org/10.1371/journal.pgph.0002216.g003

30-day mortality in children younger than five years old with severe acute malnutrition [12]. The primary outcome in this study occurred in 8.9% of patients whereas 20% of the patients in the primary study experienced treatment failure (Fig 4).

Fig 5 shows that our model achieved an AUC of 0.718 with a calibration-in-the-large slope of 0.79 (95% CI: 0.27, 1.36) and a calibration intercept of -0.68 (95% CI: -1.24, -0.19). A perfectly calibrated predictive model would have a slope of 1 and an intercept of 0. A negative

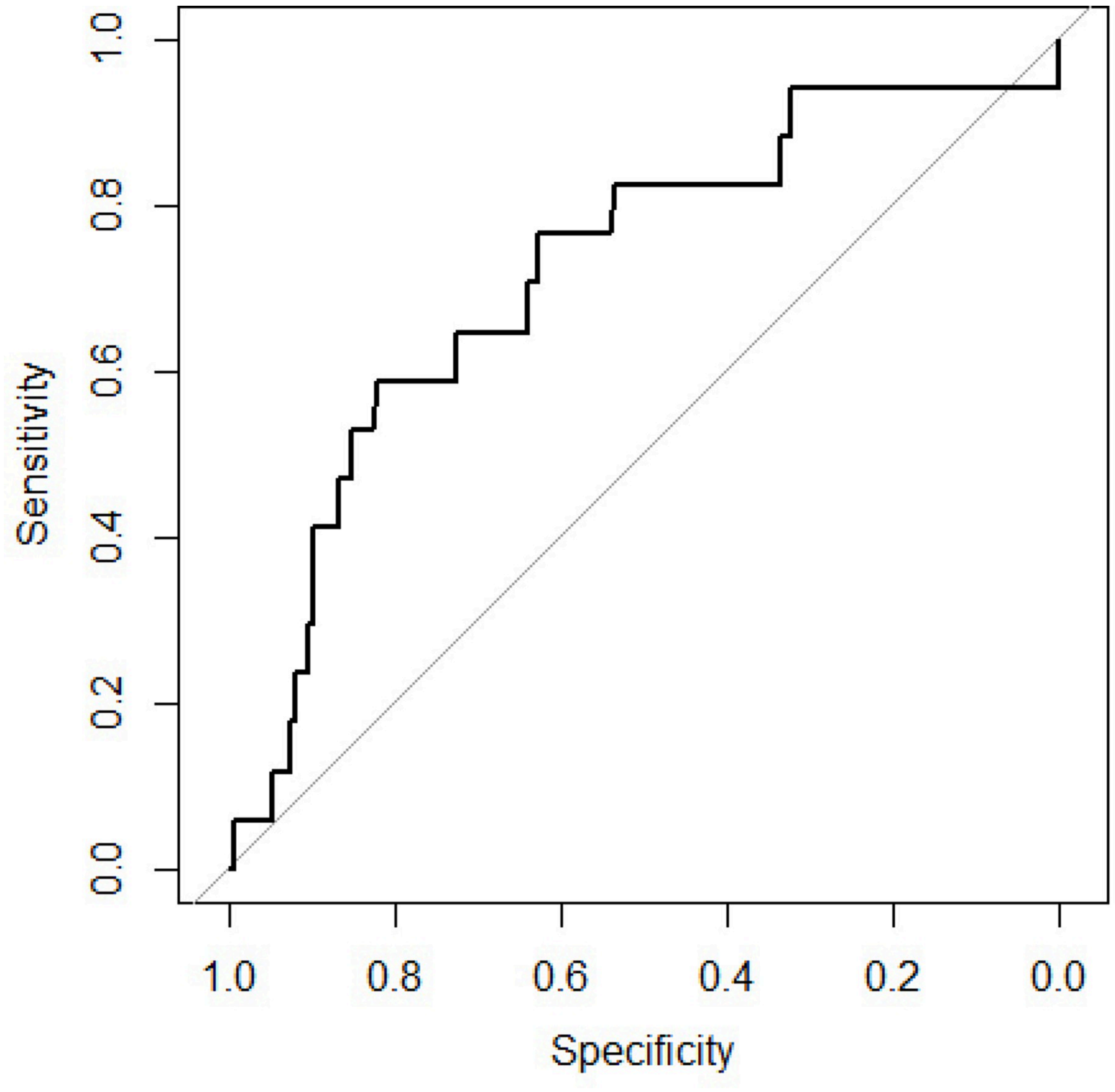

Fig 4. Receiver operating curve of the six-predictor model applied to an external dataset.

https://doi.org/10.1371/journal.pgph.0002216.g004

intercept suggests an overestimation of the risk of treatment failure [16]. A slope that is smaller than 1 suggests that the estimated probabilities of our model are too extreme, meaning that the calculated probabilities of treatment failure are too high for those at high risk of treatment failure and vice versa. A slope less than 1 may be due to overfitting of the model, though overfitting would tend to decrease discriminatory performance as well [15].

Discussion

Our study used data from a randomized control trial of 308 children between the ages of 2 and 59 months in 2018–2019 to derive a prognostic model for severe pneumonia using machine learning methods. We built a parsimonious model of six predictors, which was externally validated by a dataset that was temporally distinct, achieving a modest discriminatory performance with an AUC of 0.718. Development of clinical prediction models such as ours may assist in triaging patients and resources in the management of severe pneumonia in LMICs.

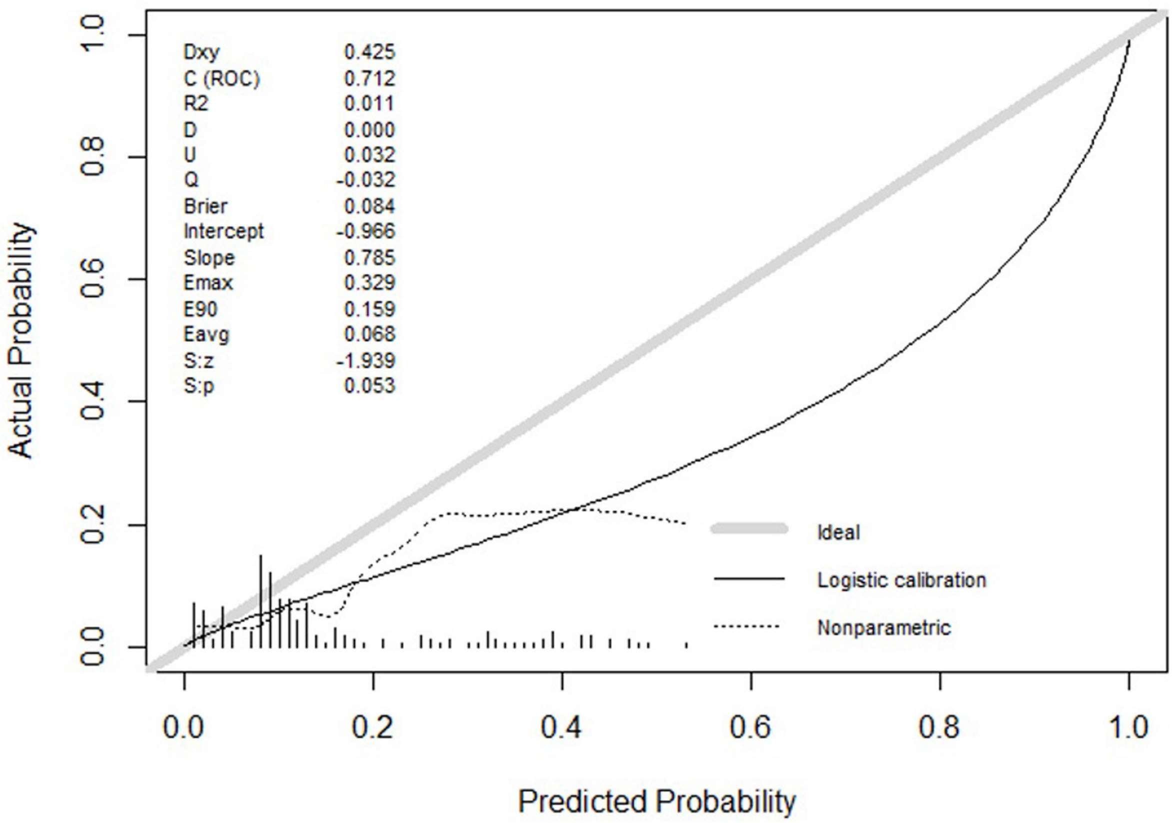

Fig 5. Calibration curve of the model by external validation. Calibration intercept = - 0.68 (95% CI: -1.24, -0.19). Calibration slope = 0.79 (95% CI: 0.27, 1.36).

https://doi.org/10.1371/journal.pgph.0002216.g005

The top six predictors in our model were, in order of importance: the presence of grunting, room air saturation, temperature, the presence of lower chest wall indrawing, the presence of respiratory distress, and the presence of central cyanosis. The addition of one more variable to our model would have modestly improved the AUC but for the sake of pragmatism, a model of six predictors was chosen, achieving an AUC of 0.6911. There is some collinearity in the predictors of our model as they focus on the respiratory system. However, the presence or absence of collinearity in a model has not been demonstrated to affect predictive outcomes [17]. Indeed, a random forest model that excluded collinear predictors such as severe respiratory distress and central cyanosis achieved a similar AUC of 0.6945. Prior studies have demonstrated that hypoxemia, central cyanosis, tachypnea, and acute malnutrition were associated with treatment failure [7, 18]. This is in line with our findings. Measures of developmental status such as mid-upper arm circumference and adjusted weight for age were also in our top 10 most important predictors.

Our work builds upon this body of knowledge by providing a model that combines these variables into a prognostic prediction rule that could be used for decision support in severe pediatric pneumonia. Our model is both parsimonious and pragmatic allowing it to be especially useful in LMIC settings. The variables screened are able to be assessed by history and physical as soon as a patient presents to medical care, potentially obviating the need for more advanced and costly diagnostics. Furthermore, several of the variables, such as room air saturation and temperature, can be assessed by non-physician support staff with minimal clinical training. Patients at high risk of treatment failure could immediately be triaged to wards with

higher levels of nursing care better able to provide more frequent monitoring and essential supportive cares such as aggressive secretion management.

Our model could be easily operationalized by incorporation into the electronic medical record or as mobile applications. A similar alert system using anthropometric measurements and the CURB-65 score is currently being used at the study site though our model would be more specific to the pediatric population. As a clinical support tool, our model can assist clinicians by identifying patients who would benefit from use of limited diagnostic resources to assess for alternative etiologies of respiratory failure as well as those who may benefit from more advanced respiratory support devices. Our tool would not necessarily guide antibiotic selection or escalation as the derivation study was not designed to assess for antibiotic resistance or other microbiologic factors as a cause of treatment failure. It may, however, suggest a patient population who would benefit from diagnostic testing in the form of blood or sputum cultures to ensure that they receive appropriate antibiotics. In sum, our model could be easily incorporated into the healthcare systems of LMIC and provide high value care by identifying high risk patients, thereby maximizing efficient use of diagnostic resources, support staff, respiratory devices, and intensive care units.

Our study has several limitations. First, our model was derived from data collected from a single-center study of hospitalized children in Bangladesh. Though the derivation study enrolled children younger than 5 years of age, it may be most applicable to younger populations, as the median age was around 7 months with an interquartile range of 6.5 months. It is likely that many of the top predictors identified would be generalizable other settings as they have been identified by other studies conducted in other LMICs to be associated with treatment failure. Secondly, some of the predictors used in our model, such as the presence or absence of respiratory distress, are subjective and may depend on the experience of the clinician. We do not have data regarding an interobserver agreement for these predictors. Thirdly, our external validation was performed using data from the same center, albeit from an earlier period. However, to our knowledge, the validation dataset was the most appropriate dataset with openly accessible data, as it examined similarly aged children with severe pneumonia as defined by the most recent guidelines for pediatric pneumonia put forth by the WHO in 2014. Though the discriminatory performance of our model was actually improved with external validation, the calibration slope and intercept suggest that our model may be systematically extreme and that the calculated probabilities of treatment failure may be too high for those at high risk of treatment failure and vice versa. In addition, our negative calibration intercept suggests that our model may overestimate the risk of treatment failure. This may be due to the higher incidence of treatment failure in the primary dataset than in the external validation dataset. In addition, the derivation study found that the amoxicillin treatment was associated with a reduction in treatment failure compared with the ampicillin arm. We removed the treatment arm as a predictor in this study to make our model more generalizable, though this potentially reduces its predictive performance. Finally, the sample sizes of our studies for the derivation and validation of our clinical prediction rule were relatively small, at 308 and 191 patients, respectively, resulting in relatively large standard deviations in our AUC.

Conclusions

We used random forest models to develop a parsimonious, 6-predictor model for treatment failure in children aged 2–59 months with severe pneumonia, achieving an externally validated AUC of 0.718. We identified grunting, room air saturation, temperature, lower chest wall indrawing, respiratory distress, and central cyanosis as the most important predictors of treatment failure. Future studies should reproduce our methods in other centers with the aim of

developing and validating clinical prediction rules that may help improve patient care and triage healthcare resources in diverse global settings.

Supporting information

S1 Text. TRIPOD checklist.
(DOCX)

S1 Data. De-identified derivation dataset.
(XLSX)

S2 Data. De-identified external validation dataset.
(XLSX)

Acknowledgments

We gratefully acknowledge core donors of the International Centre for Diarrhoeal Disease Research, Bangladesh for their support and commitment to our research efforts. The International Centre for Diarrhoeal Disease Research, Bangladesh receives unrestricted support from the Government of the People's Republic of Bangladesh, Global Affairs Canada, the Swedish International Development Cooperation Agency, and the UK Department for International Development. We would like to express our sincere thanks to all clinical fellows, nurses, and hospital support staff for their invaluable support and contribution to patient care.

Author Contributions

Conceptualization: Gazi Md. Salahuddin Mamun, Ben J. Brintz, Mohammod Jobayer Chisti, Daniel T. Leung, Lubaba Shahrin.

Data curation: Michael Zou, Abu Sayem Mirza Md. Hasibur Rahman.

Formal analysis: Michael Zou, Ben J. Brintz.

Investigation: Monira Sarmin, Abu Sayem Mirza Md. Hasibur Rahman, Mst Mahmuda Ackhter.

Methodology: Gazi Md. Salahuddin Mamun, Michael Zou, Irin Parvin, Daniel T. Leung, Lubaba Shahrin.

Project administration: Abu Sayem Mirza Md. Hasibur Rahman, Irin Parvin, Lubaba Shahrin.

Resources: Mst Mahmuda Ackhter, Daniel T. Leung, Lubaba Shahrin.

Software: Mst Mahmuda Ackhter.

Supervision: Ben J. Brintz, Mohammod Jobayer Chisti, Daniel T. Leung, Lubaba Shahrin.

Visualization: Michael Zou.

Writing – original draft: Gazi Md. Salahuddin Mamun, Michael Zou, Monira Sarmin, Irin Parvin, Mohammod Jobayer Chisti, Daniel T. Leung, Lubaba Shahrin.

Writing – review & editing: Gazi Md. Salahuddin Mamun, Monira Sarmin, Mohammod Jobayer Chisti, Daniel T. Leung, Lubaba Shahrin.

References

1. Organization WH. WHO methods and data sources for country-level causes of death 2000–2019. Global health estimates technical paper WHO/DDI/DNA/GHE/20202 Geneva: World Health Organization. 2020.
2. UNICEF, editor UNICEF Analysis Based on WHO and Maternal and Child Epidemiology Estimation Group Interim Estimates Produced in September 2019, Applying Cause Fractions for the Year 2017 to United Nations Inter-Agency Group for Child Mortality Estimation Estimates for the Year 2018. Convention on the Rights of the Child; 2019.
3. Organization WH. Stakeholder consultative meeting on prevention and management of childhood pneumonia and diarrhoea: report, 12–14 October 2021. 2022.
4. Lassi ZS, Imdad A, Bhutta ZA. Short-course versus long-course intravenous therapy with the same antibiotic for severe community-acquired pneumonia in children aged two months to 59 months. Cochrane Database of Systematic Reviews. 2017;(10).
5. Same RG, Amoah J, Hsu AJ, Hersh AL, Sklansky DJ, Cosgrove SE, et al. The association of antibiotic duration with successful treatment of community-acquired pneumonia in children. Journal of the Pediatric Infectious Diseases Society. 2021; 10(3):267–73. https://doi.org/10.1093/jpids/piaa055 PMID: 32525203
6. Chisti MJ, Salam MA, Smith JH, Ahmed T, Pietroni MA, Shahunja K, et al. Bubble continuous positive airway pressure for children with severe pneumonia and hypoxaemia in Bangladesh: an open, randomised controlled trial. The Lancet. 2015; 386(9998):1057–65. https://doi.org/10.1016/S0140-6736(15)60249-5 PMID: 26296950
7. Muro RP, Masoza TS, Kasanga G, Kayange N, Kidenya BR. Predictors and outcome of first line treatment failure among under-five children with community acquired severe pneumonia at Bugando Medical Centre, Mwanza, Tanzania: A prospective cohort study. PloS one. 2020; 15(12):e0243636. https://doi.org/10.1371/journal.pone.0243636 PMID: 33306722
8. Chisti MJ, Salam MA, Bardhan PK, Faruque AS, Shahid AS, Shahunja K, et al. Treatment failure and mortality amongst children with severe acute malnutrition presenting with cough or respiratory difficulty and radiological pneumonia. PLoS One. 2015; 10(10):e0140327. https://doi.org/10.1371/journal.pone.0140327 PMID: 26451603
9. Shahrin L, Chisti MJ, Sarmin M, Rahman ASMM, Shahid ASMSB, Islam M, et al. Intravenous Amoxicillin Plus Intravenous Gentamicin for Children with Severe Pneumonia in Bangladesh: An Open-Label, Randomized, Non-Inferiority Controlled Trial. Life. 2021; 11(12):1299. https://doi.org/10.3390/life11121299 PMID: 34947830
10. Shahrin L, Chisti MJ, Shahid ASMSB, Rahman ASMMH, Islam MZ, Afroze F, et al. Injectable amoxicillin versus injectable ampicillin plus gentamicin in the treatment of severe pneumonia in children aged 2 to 59 months: protocol for an open-label randomized controlled trial. JMIR research protocols. 2020; 9(11):e17735. https://doi.org/10.2196/17735 PMID: 33136058
11. Organization WH. Pocket book of hospital care for children: guidelines for the management of common childhood illnesses: World Health Organization; 2013.
12. Shahrin L, Chisti MJ, Brintz B, Islam Z, Shahid AS, Hassan MZ, et al. Clinical and laboratory predictors of 30-day mortality in severe acute malnourished children with severe pneumonia. Tropical Medicine & International Health. 2020; 25(11):1422–30. https://doi.org/10.1111/tmi.13484 PMID: 32985047
13. Van Buuren S, Groothuis-Oudshoorn K. mice: Multivariate imputation by chained equations in R. Journal of statistical software. 2011; 45:1–67.
14. Debeer D, Hothorn T, Strobl C, Debeer MD. Package 'permimp'. 2021.
15. Van Calster B, McLernon DJ, Van Smeden M, Wynants L, Steyerberg EW. Calibration: the Achilles heel of predictive analytics. BMC medicine. 2019; 17(1):1–7.
16. Steyerberg EW, Vickers AJ, Cook NR, Gerds T, Gonen M, Obuchowski N, et al. Assessing the performance of prediction models: a framework for some traditional and novel measures. Epidemiology (Cambridge, Mass). 2010; 21(1):128.
17. Leeuwenberg AM, van Smeden M, Langendijk JA, van der Schaaf A, Mauer ME, Moons KG, et al. Performance of binary prediction models in high-correlation low-dimensional settings: a comparison of methods. Diagnostic and prognostic research. 2022; 6(1):1. https://doi.org/10.1186/s41512-021-00115-5 PMID: 35016734
18. Addo-Yobo E, Chisaka N, Hassan M, Hibberd P, Lozano JM, Jeena P, et al. Oral amoxicillin versus injectable penicillin for severe pneumonia in children aged 3 to 59 months: a randomised multicentre equivalency study. The Lancet. 2004; 364(9440):1141–8. https://doi.org/10.1016/S0140-6736(04)17100-6 PMID: 15451221
